# Temporal Trends of Adults Gaining or Losing Enrolment in Primary Care Medical Homes in Ontario, Canada: A Retrospective Serial Cohort Analysis

**DOI:** 10.7759/cureus.110315

**Published:** 2026-06-05

**Authors:** Kamila Premji, Michael E Green, Paul Nguyen, Maria Mathews, Lise Bjerre, Jonathan Fitzsimon, Jennifer Boyle, Antoine St-Amant, Eliot Frymire, Leanda Godfrey, Richard H Glazier

**Affiliations:** 1 Family Medicine, Institut du Savoir Montfort, Ottawa, CAN; 2 Family Medicine, Bruyere Health Research Institute, Ottawa, CAN; 3 Family Medicine, University of Ottawa, Ottawa, CAN; 4 Family Medicine, NOSM University, Ottawa, CAN; 5 Biostatistics, Institute for Clinical Evaluative Sciences, Kingston, CAN; 6 Family Medicine, Western University, London, CAN; 7 Patient Expertise in Research Collaboration, McMaster University, Hamilton, CAN; 8 Epidemiology, University of Ottawa, Ottawa, CAN; 9 Health Services and Policy Research, Queen's University, Kingston, CAN; 10 Family Medicine, University of Toronto, Toronto, CAN

**Keywords:** health workforce policy, patient centered medical home, patient enrolment models, primary care attachment, regular primary care provider

## Abstract

Introduction

Patient attachment to a primary care medical home has emerged as a key strategy to strengthen population health in high-income countries, including the U.S., Canada, New Zealand, the United Kingdom, and other European countries. In Canada, Ontario introduced primary care medical homes beginning in 2002, with capitation-based and interdisciplinary team-based models becoming increasingly prominent over time. Citing cost concerns, however, various government policies were subsequently introduced to limit physician entry into these types of medical homes. Entry into medical homes, remunerated mainly by fee-for-service and without funding for interdisciplinary teams, remained unrestricted. We sought to examine the dynamics of patients’ attachment to a primary care medical home amidst this shifting policy landscape.

Methods

We conducted a retrospective, population-level serial cohort analysis using linked health administrative datasets. We examined the monthly proportion of Ontario adults gaining or losing enrolment in a primary care medical home from 2009 to 2021. Temporal trends in net monthly enrolment gains/losses were assessed using linear regression, with statistical significance evaluated using a generalized additive mixed model accounting for temporal autocorrelation. The number of medical home physicians per capita over time was also examined. Unadjusted cross-sectional descriptive analyses at three time points (2009, 2014, and 2021) were conducted to characterize the sociodemographic profiles of adults gaining or losing enrolment.

Results

Initially, a larger proportion of adults were gaining rather than losing enrolment, but this gap narrowed over time. Medical home enrolment peaked at 77.3% (May 2015) and subsequently declined despite growth in physicians per capita practicing in medical homes. There was an overall statistically significant decline in net enrolment rates. After losing enrolment, patients experienced long durations without formal enrolment in a medical home (mean 57.5 months (SD 44.7)). Enrolment losses appeared to occur predominantly from fee-for-service-based medical homes and disproportionately affected equity-deserving populations.

Conclusion

Ontario’s primary care medical home program did not achieve full population coverage, and policy changes limiting physician entry into capitation-based and interdisciplinary team-based medical homes occurred alongside a steady erosion in net enrolments. These dynamics may have impeded the full realization of the benefits of medical home enrolment at the health system level. Recent policy initiatives that remove previously implemented barriers to physician entry into capitation and team-based medical homes may help reverse this trend.

## Introduction

Patient attachment to a primary care medical home has emerged as a key strategy for strengthening population health in high-income countries [1-5]. Medical homes establish a formal relationship between the patient and the medical home’s practitioners through “enrolment” (also termed “rostering,” or “empanelment”), and serve as the main source of primary care while gatekeeping and coordinating access to other services [2,6]. The objectives of the medical home model include improving health outcomes, enhancing patient satisfaction, and reducing health system costs by optimizing continuity of care, facilitating appropriate use of resources, enabling systematic preventive and chronic disease care, and enabling system-level primary care performance measurement and quality improvement [6-9]. 

In Canada, family physicians provide most primary care services [10]. Ontario, the country’s most populous province, has led the implementation of the medical home model in Canada, with voluntary family physician uptake of the model beginning in 2002 [11]. Ontario’s medical homes require physicians to work in larger groups instead of the traditional solo or duo office arrangement, sometimes with additional funding to include interdisciplinary professionals [11]. Physician payment reform has also been a key aspect of Ontario’s medical home model. This has involved moving physicians from traditional fee-for-service remuneration to either capitation-based or “enhanced fee-for-service” payment, the former being comprised mainly of a fixed annual payment per enrolled patient regardless of care sought, and the latter being comprised mainly of fee-for-service payments with additional bonuses for activities such as after-hours care and preventive care [11]. Enhanced fee-for-service medical homes were not eligible for additional funding for interdisciplinary professionals [11].

Associated with higher financial and work satisfaction for physicians compared with enhanced fee-for-service and traditional fee-for-service practices, by 2011, medical homes linked to capitation payments or teams became the most widely adopted medical home models [12-14]. This trend coincided with a growing family physician workforce and increased patient-reported primary care attachment [15]. Ontario-based research shows patients enrolled in medical homes have fewer emergency department (ED) visits [16], higher cancer screening rates [17], and better diabetes management [18]. However, in 2012, the Ontario government began limiting physician entry into medical homes with capitation-based remuneration and interdisciplinary teams due to cost concerns, with the strictest limits being implemented in 2015 and lasting nearly a decade [19,20]. Entry into medical homes remunerated by enhanced fee-for-service payments remained unrestricted [19,20].

Amidst policy changes affecting physician participation in Ontario’s primary care medical home models, we sought to examine population-level temporal trends in adults gaining or losing formal medical home enrolment between January 2009 and December 2021. 

## Materials and methods

We conducted a retrospective serial cohort analysis of health administrative datasets that were linked using unique, encoded identifiers and analyzed at ICES. ICES, formerly known as the Institute for Clinical Evaluative Sciences, is an independent, non-profit research institute whose legal status under Ontario’s health information privacy law allows it to collect and analyze healthcare and demographic data without consent, for health system evaluation and improvement.

We identified monthly cohorts of Ontario adults gaining or losing formal enrolment in a primary care medical home between 2009 and 2021. We limited our analysis to adults because approximately a quarter of Ontario children receive primary care from pediatricians [21]. The index date was defined as the last date of the month within which a patient was enrolled in or de-enrolled from a medical home. We excluded adults without Ontario Health Insurance Plan (OHIP) coverage, those residing outside Ontario at the index date, and those who died before the index date. Patients rostered to Community Health Groups, which evolved into a non-rostering model known as Community Health Centers (CHCs) during our study period, were also excluded to avoid misclassifying their transition as a loss of medical home enrolment. To address other administrative issues causing lapses in medical home enrolment that did not represent a “true loss” of enrolment, patients were considered continuously enrolled if: i) the lapse between enrolment periods was less than six months; or ii) they subsequently re-enrolled with the same physician or group, or had OHIP billings from the same physician within two years following the index date. These criteria align with evidence from the U.S. and Switzerland around when health system effects begin to occur after losing primary care attachment [22,23]. Similarly, a patient was considered to have had a “true gain” in medical home enrolment if they were not enrolled in the month before the index date and not enrolled with the same physician or group in the two years before the index date.

Monthly gain and loss rates were calculated using all Ontario residents and all Ontario adult residents at each monthly index date as the denominator, and net monthly changes in enrolment were derived as the difference between gains and losses. To evaluate temporal trends in net enrolment over time, we fitted a linear regression model with time (in months) as a continuous independent variable, providing an estimate of the overall direction and magnitude of change across the study period. Higher-order polynomial regression models were examined and yielded comparable results to the fitted linear model. Because the data represent repeated monthly observations with potential temporal autocorrelation, the statistical significance of the trend was evaluated with a generalized additive mixed model, which accounts for correlation between adjacent time points and provides valid inference for longitudinal time-series data.

As a secondary analysis, we conducted an unadjusted cross-sectional descriptive analysis of Ontario adults at three time points (March 31, 2009, March 31, 2014, and March 31, 2021) using enrolment status to understand the sociodemographic characteristics of individuals gaining or losing enrolment in a primary care medical home. These analyses were descriptive in nature with no formal hypothesis testing performed, as the objective was to summarize population characteristics rather than assess group differences.

Detailed data source and covariate definitions are provided in Appendix A. 

We used SAS software version 9.4 (SAS Institute Inc., Cary, USA) for all analyses. Figures were generated using Microsoft Excel (Microsoft Corporation, Redmond, USA).

The use of health administrative data in this study was authorized under section 45 of Ontario’s Personal Health Information Protection Act (PHIPA). Therefore, research ethics board approval and informed consent were not required.

## Results

**Figure 1:**
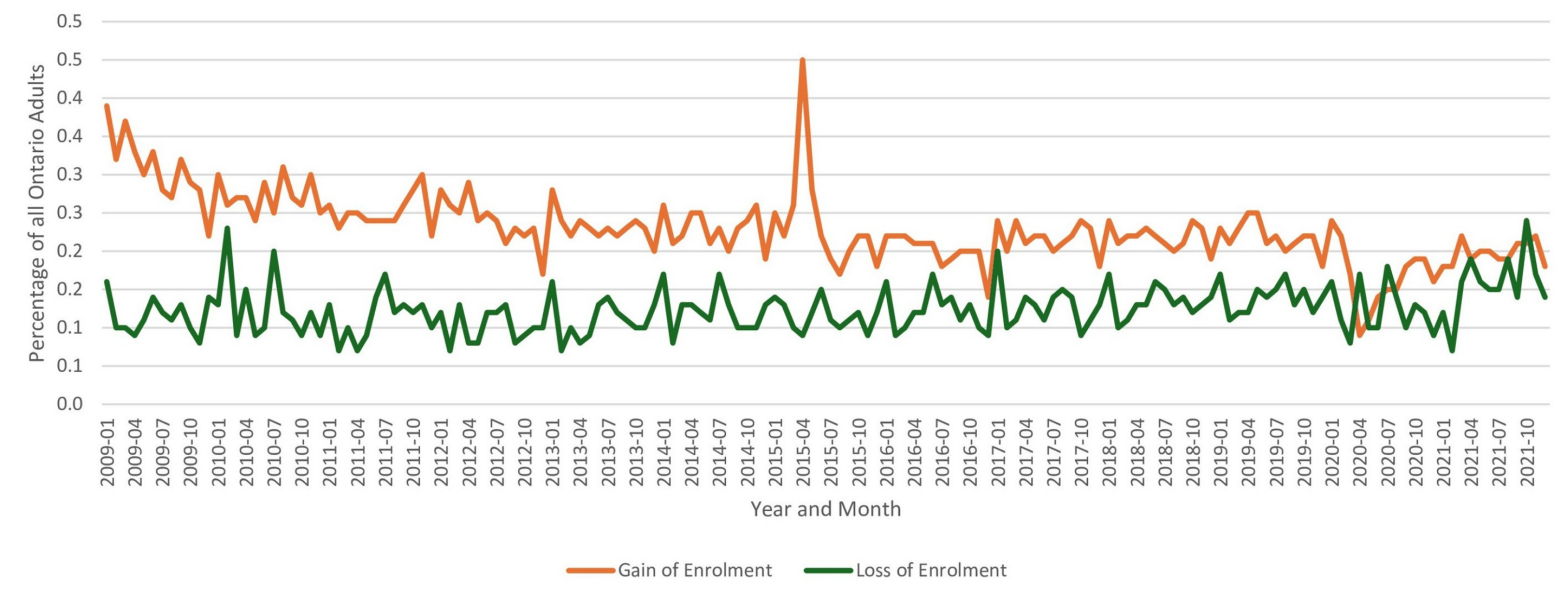
Monthly gains and losses in enrolment over time

**Figure 2:**
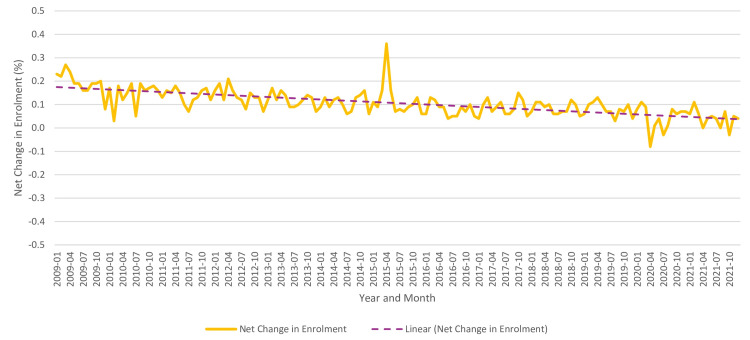
Net changes in enrolment over time

**Figure 3:**
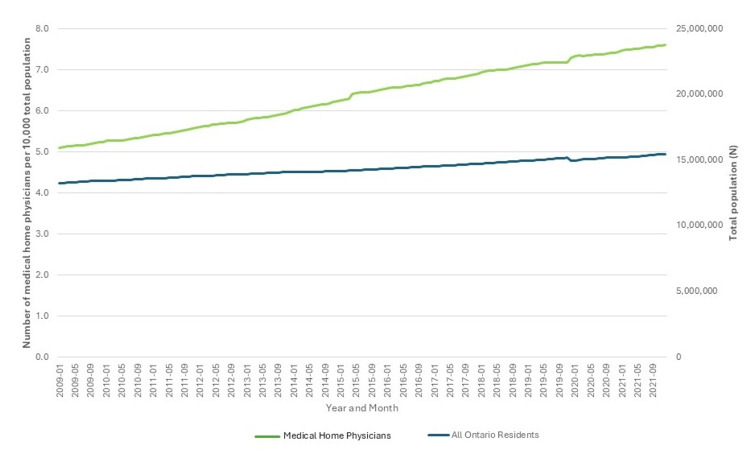
Medical home physicians per capita over time compared with population growth

**Figure 4:**
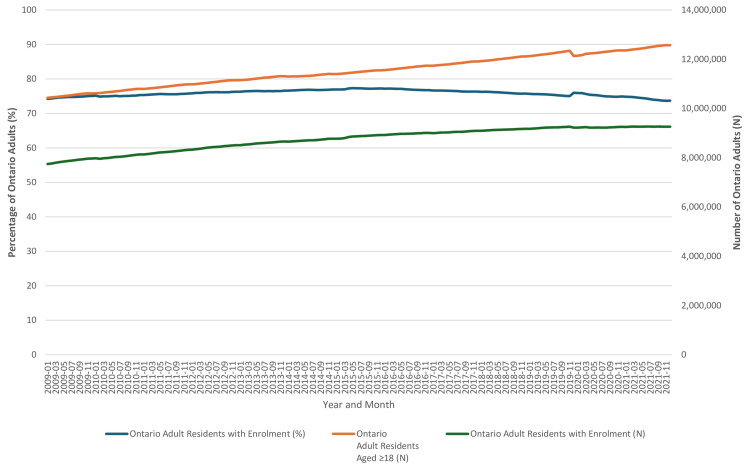
Ontario adults enrolled in a medical home over time (% and N) compared with population growth (N)

The total number of de-enrolments during the study period (2009-2021) was 2,225,719 (monthly mean 14,267 (SD 3,853)), and the total number of enrolments was 4,100,919 (monthly mean 26,288 (SD 4,442)). Initially, a larger proportion of the population gained rather than lost enrolment (Figure 1). This gap, however, narrowed over the study period (Figure 1), with the total yearly gain in enrolment declining from 3.70% in 2009 to 2.37% in 2021. The slope of the monthly trend in net changes in enrolment was -0.0009% (or 0.9 per 100,000) and was statistically significant (p<0.001) (Figure 2). Alongside population growth from 13,250,882 persons (10,434,097 adults) to 15,430,758 persons (12,570,561 adults) over the study period, the number of physicians per capita practicing in a medical home grew from 5.09 per 10,000 persons (6.47 per 10,000 adults) to 7.61 per 10,000 persons (9.34 per 10,000 adults) (Figure 3). However, the overall proportion of adults with enrolment declined after 2015 (Figure 4). A spike in net enrolments and medical home physicians per capita occurred in April 2015, and dips in these measures occurred in April 2020 (Figure 1 and Figure 3). The mean and median durations of unattachment following a first loss of enrolment were 57.5 months (SD 44.7) and 44 months (IQR 23-82), respectively. Expanded versions of each figure can be found in Appendix B.

After excluding those under 18 years of age, those with out-of-province residency, long-term care home residents, and those using community health centres or community health groups, the population of Ontario adults at our three timepoints for cross-sectional analysis was 10,125,062 (March 31, 2009), 10,534,625 (March 31, 2014), and 11,560,073 (March 31, 2021). Table 1, Table 2, and Table 3 describe the characteristics of those enrolled and not enrolled in a medical home at the three time points. An increasing proportion of medical home patients were enrolled in capitation-based medical homes over time, while losses in enrolment predominantly occurred in medical homes remunerated through enhanced fee-for-service payments. Compared with males and younger adults, females and older adults had consistently higher rates of enrolment throughout. Gaps in enrolment for equity-deserving populations persisted throughout the study period, with declining enrolment rates after 2014 disproportionately affecting newcomers, racialized populations, patients with a mental health diagnosis in the previous two years, small town residents (Rurality Index of Ontario score 10-39), and patients in the lowest income and material resources quintiles. By the end of the study period, we saw declines in enrolment for those with the highest overall comorbidity scores, though for certain individual chronic conditions (chronic obstructive pulmonary disease (COPD), congestive heart failure (CHF), hypertension, diabetes, dementia), enrolment rates improved. Compared to those with enrolment, the proportion with any ED visit was lower among those without enrolment, but among ED users, the mean number of ED visits was higher for those without enrolment.

**Table 1:** Sociodemographic characteristics by enrolment status for Ontario adults over time Percentages are column percentages. Abbreviations and definitions: RIO, Rurality Index of Ontario; Urban, RIO score 0-9; Small town, RIO score 10-39; Rural, RIO score 40+

	Ontario Adult Population								
	2009			2014			2021		
Variable	Total	Enrolled in a medical home	Not enrolled in a medical home	Total	Enrolled in a medical home	Not enrolled in a medical home	Total	Enrolled in a medical home	Not enrolled in a medical home
Overall	10,125,062 (100%)	7,038,103 (69.5%)	3,086,959 (30.5%)	10,534,625 (100%)	8,243,715 (78.3%)	2,290,910 (21.8%)	11,560,073 (100%)	8,944,621 (77.4%)	2,615,452 (22.6%)
Mean Age: Years (SD)	46.48 (17.55)	48.07 (17.68)	42.85 (16.68)	47.48 (17.94)	48.58 (18.01)	43.52 (17.13)	48.67 (18.42)	50.16 (18.45)	43.59 (17.38)
Median Age: Years (Q1-Q3)	45 (32-59)	47 (34-61)	41 (29-54)	47 (33-60)	49 (34-62)	42 (29-55)	48 (33-63)	50 (35-64)	40 (29-56)
Age group: 18-34 years	2,910,123 (28.7%)	1,787,300 (25.4%)	1,122,823 (36.4%)	2,978,025 (28.3%)	2,133,091 (25.9%)	844,934 (36.9%)	3,197,849 (27.7%)	2,194,219 (24.5%)	1,003,630 (38.4%)
Age group: 35-49 years	3,032,155 (29.9%)	2,068,405 (29.4%)	963,750 (31.2%)	2,793,654 (26.5%)	2,158,676 (26.2%)	634,978 (27.7%)	2,853,686 (24.7%)	2,160,988 (24.2%)	692,698 (26.5%)
Age group: 50-64 years	2,485,461 (24.5%)	1,836,140 (26.1%)	649,321 (21.0%)	2,774,994 (26.3%)	2,258,098 (27.4%)	516,896 (22.6%)	2,968,530 (25.7%)	2,412,565 (27.0%)	555,965 (21.3%)
Age group: 65-79 years	1,269,846 (12.5%)	1,006,089 (14.3%)	263,757 (8.5%)	1,491,322 (14.2%)	1,268,219 (15.4%)	223,103 (9.7%)	1,927,183 (16.7%)	1,647,327 (18.4%)	279,856 (10.7%)
Age group: 80+ years	427,477 (4.2%)	340,169 (4.8%)	87,308 (2.8%)	496,630 (4.7%)	425,631 (5.2%)	70,999 (3.1%)	612,825 (5.3%)	529,522 (5.9%)	83,303 (3.2%)
Sex: Male	4,931,303 (48.7%)	3,195,731 (45.4%)	1,735,572 (56.2%)	5,129,668 (48.7%)	3,845,158 (46.6%)	1,284,510 (56.1%)	5,669,597 (49.0%)	4,214,186 (47.1%)	1,455,411 (55.6%)
Sex: Female	5,193,759 (51.3%)	3,842,372 (54.6%)	1,351,387 (43.8%)	5,404,957 (51.3%)	4,398,557 (53.4%)	1,006,400 (43.9%)	5,890,476 (51.0%)	4,730,435 (52.9%)	1,160,041 (44.4%)
Rurality (RIO): Urban	7,316,270 (72.3%)	4,964,195 (70.5%)	2,352,075 (76.2%)	7,668,632 (72.8%)	5,910,505 (71.7%)	1,758,127 (76.7%)	8,442,965 (73.0%)	6,439,702 (72.0%)	2,003,263 (76.6%)
Rurality (RIO): Small town	1,955,590 (19.3%)	1,495,372 (21.2%)	460,218 (14.9%)	2,015,292 (19.1%)	1,674,012 (20.3%)	341,280 (14.9%)	2,189,249 (18.9%)	1,788,471 (20.0%)	400,778 (15.3%)
Rurality (RIO): Rural	768,882 (7.6%)	534,938 (7.6%)	233,944 (7.6%)	767,739 (7.3%)	609,907 (7.4%)	157,832 (6.9%)	802,038 (6.9%)	641,622 (7.2%)	160,416 (6.1%)
Rurality (RIO): Missing	84,320 (0.8%)	43,598 (0.6%)	40,722 (1.3%)	82,962 (0.8%)	49,291 (0.6%)	33,671 (1.5%)	125,821 (1.1%)	74,826 (0.8%)	50,995 (1.9%)
Income quintile: 1 (Lowest)	1,940,789 (19.2%)	1,237,934 (17.6%)	702,855 (22.9%)	2,085,055 (19.8%)	1,523,071 (18.5%)	561,984 (24.6%)	2,198,088 (19.1%)	1,567,736 (17.6%)	630,352 (24.2%)
Income quintile: 2	2,008,654 (19.9%)	1,373,257 (19.6%)	635,397 (20.7%)	2,120,986 (20.2%)	1,635,513 (19.9%)	485,473 (21.2%)	2,281,570 (19.8%)	1,732,941 (19.4%)	548,629 (21.1%)
Income quintile: 3	2,010,984 (19.9%)	1,418,413 (20.2%)	592,571 (19.3%)	2,107,264 (20.0%)	1,667,820 (20.3%)	439,444 (19.2%)	2,338,407 (20.3%)	1,832,086 (20.5%)	506,321 (19.4%)
Income quintile: 4	2,080,353 (20.6%)	1,504,300 (21.4%)	576,053 (18.8%)	2,063,747 (19.6%)	1,667,187 (20.3%)	396,560 (17.4%)	2,343,803 (20.3%)	1,877,138 (21.0%)	466,665 (17.9%)
Income quintile: 5 (Highest)	2,046,492 (20.3%)	1,484,469 (21.2%)	562,023 (18.3%)	2,139,320 (20.3%)	1,737,939 (21.1%)	401,381 (17.6%)	2,365,129 (20.5%)	1,912,927 (21.4%)	452,202 (17.4%)
Material resources quintile: 1 (Least marginalized)	1,830,487 (18.2%)	1,370,722 (19.6%)	459,765 (15.1%)	2,280,089 (21.8%)	1,834,304 (22.4%)	445,785 (19.8%)	2,391,464 (20.8%)	1,900,306 (21.4%)	491,158 (19.1%)
Material resources quintile: 2	1,913,917 (19.0%)	1,393,033 (19.9%)	520,884 (17.1%)	2,183,006 (20.9%)	1,762,061 (21.5%)	420,945 (18.7%)	2,526,228 (22.0%)	2,015,338 (22.7%)	510,890 (19.8%)
Material resources quintile: 3	1,957,267 (19.4%)	1,386,703 (19.8%)	570,564 (18.7%)	2,028,402 (19.4%)	1,607,022 (19.6%)	421,380 (18.7%)	2,360,854 (20.6%)	1,856,037 (20.9%)	504,817 (19.6%)
Material resources quintile: 4	2,071,229 (20.6%)	1,409,038 (20.1%)	662,191 (21.7%)	1,980,366 (19.0%)	1,530,310 (18.7%)	450,056 (20.0%)	2,099,082 (18.3%)	1,597,602 (18.0%)	501,480 (19.5%)
Material resources quintile: 5 (Most marginalized)	2,291,683 (22.8%)	1,451,289 (20.7%)	840,394 (27.5%)	1,968,316 (18.9%)	1,454,170 (17.8%)	514,146 (22.8%)	2,095,581 (18.3%)	1,526,652 (17.2%)	568,929 (22.1%)
Racialized and newcomer populations quintile: 1 (Least marginalized)	1,688,416 (16.8%)	1,234,943 (17.6%)	453,473 (14.8%)	1,700,895 (16.3%)	1,384,783 (16.9%)	316,112 (14.0%)	1,770,071 (15.4%)	1,429,505 (16.1%)	340,566 (13.2%)
Racialized and newcomer populations quintile: 2	1,746,392 (17.4%)	1,307,809 (18.7%)	438,583 (14.4%)	1,790,001 (17.1%)	1,460,887 (17.8%)	329,114 (14.6%)	1,941,748 (16.9%)	1,563,698 (17.6%)	378,050 (14.7%)
Racialized and newcomer populations quintile: 3	1,859,628 (18.5%)	1,361,551 (19.4%)	498,077 (16.3%)	1,904,356 (18.2%)	1,512,738 (18.5%)	391,618 (17.4%)	2,043,430 (17.8%)	1,606,505 (18.1%)	436,925 (17.0%)
Racialized and newcomer populations quintile: 4	2,048,019 (20.3%)	1,388,871 (19.8%)	659,148 (21.6%)	2,187,933 (21.0%)	1,688,185 (20.6%)	499,748 (22.2%)	2,456,665 (21.4%)	1,885,134 (21.2%)	571,531 (22.2%)
Racialized and newcomer populations quintile: 5 (Most marginalized)	2,722,128 (27.0%)	1,717,611 (24.5%)	1,004,517 (32.9%)	2,856,994 (27.4%)	2,141,274 (26.2%)	715,720 (31.8%)	3,261,295 (28.4%)	2,411,093 (27.1%)	850,202 (33.0%)
New arrival to Ontario: No	8,924,761 (88.1%)	6,390,482 (90.8%)	2,534,279 (82.1%)	9,483,052 (90.0%)	7,579,469 (91.9%)	1,903,583 (83.1%)	10,324,859 (89.3%)	8,253,247 (92.3%)	2,071,612 (79.2%)
New arrival to Ontario: Yes	1,200,301 (11.9%)	647,621 (9.2%)	552,680 (17.9%)	1,051,573 (10.0%)	664,246 (8.1%)	387,327 (16.9%)	1,235,214 (10.7%)	691,374 (7.7%)	543,840 (20.8%)

**Table 2:** Health characteristics by enrolment status for Ontario adults over time Percentages are column percentages. Abbreviations: ADG, morbidity measure based on Aggregated Diagnosis Group; RUB, morbidity measure based on Resource Utilization Band; EFFS, enhanced fee-for-service medical home; CAP, capitation-based medical home; FHT, Family Health Team medical home; OGP, other medical home type; COPD, chronic obstructive pulmonary disease; CHF, congestive heart failure

	Ontario Adult Population								
	2009			2014			2021		
Variable	Total	Enrolled in a medical home	Not enrolled in a medical home	Total	Enrolled in a medical home	Not enrolled in a medical home	Total	Enrolled in a medical home	Not enrolled in a medical home
Overall	10,125,062 (100%)	7,038,103 (69.5%)	3,086,959 (30.5%)	10,534,625 (100%)	8,243,715 (78.3%)	2,290,910 (21.8%)	11,560,073 (100%)	8,944,621 (77.4%)	2,615,452 (22.6%)
ADG: No/low comorbidity (0-4)	4,995,474 (49.3%)	2,961,945 (42.1%)	2,033,529 (65.9%)	5,324,050 (50.5%)	3,839,332 (46.6%)	1,484,718 (64.8%)	6,320,042 (54.7%)	4,548,246 (50.8%)	1,771,796 (67.7%)
ADG: Moderate comorbidity (5-9)	3,948,335 (39.0%)	3,112,784 (44.2%)	835,551 (27.1%)	3,982,032 (37.8%)	3,356,880 (40.7%)	625,152 (27.3%)	3,980,844 (34.4%)	3,340,782 (37.3%)	640,062 (24.5%)
ADG: High comorbidity (10+)	1,181,253 (11.7%)	963,374 (13.7%)	217,879 (7.1%)	1,228,543 (11.7%)	1,047,503 (12.7%)	181,040 (7.9%)	1,259,187 (10.9%)	1,055,593 (11.8%)	203,594 (7.8%)
RUB: Non-user/healthy user (0-1)	1,544,649 (15.3%)	581,725 (8.3%)	962,924 (31.2%)	1,565,591 (14.9%)	901,524 (10.9%)	664,067 (29.0%)	2,000,006 (17.3%)	1,143,399 (12.8%)	856,607 (32.8%)
RUB: Low morbidity (2)	1,657,510 (16.4%)	1,084,544 (15.4%)	572,966 (18.6%)	1,741,722 (16.5%)	1,317,835 (16.0%)	423,887 (18.5%)	1,884,879 (16.3%)	1,431,395 (16.0%)	453,484 (17.3%)
RUB: Moderate morbidity (3)	4,960,472 (49.0%)	3,803,458 (54.0%)	1,157,014 (37.5%)	5,123,601 (48.6%)	4,246,042 (51.5%)	877,559 (38.3%)	5,319,058 (46.0%)	4,398,111 (49.2%)	920,947 (35.2%)
RUB: High morbidity (4+)	1,962,431 (19.4%)	1,568,376 (22.3%)	394,055 (12.8%)	2,103,711 (20.0%)	1,778,314 (21.6%)	325,397 (14.2%)	2,356,130 (20.4%)	1,971,716 (22.0%)	384,414 (14.7%)
Medical home model: EFFS	4,056,513 (40.1%)	4,056,513 (57.6%)	0 (0.0%)	2,990,038 (28.4%)	2,990,038 (36.3%)	0 (0.0%)	2,899,944 (25.1%)	2,899,944 (32.4%)	0 (0.0%)
Medical home model: CAP	1,373,614 (13.6%)	1,373,614 (19.5%)	0 (0.0%)	2,678,507 (25.4%)	2,678,507 (32.5%)	0 (0.0%)	3,174,275 (27.5%)	3,174,275 (35.5%)	0 (0.0%)
Medical home model: FHT	1,521,329 (15.0%)	1,521,329 (21.6%)	0 (0.0%)	2,490,324 (23.6%)	2,490,324 (30.2%)	0 (0.0%)	2,789,873 (24.1%)	2,789,873 (31.2%)	0 (0.0%)
Medical home: OGP	86,647 (0.9%)	86,647 (1.2%)	0 (0.0%)	84,846 (0.8%)	84,846 (1.0%)	0 (0.0%)	80,529 (0.7%)	80,529 (0.9%)	0 (0.0%)
Medical home: Receiving regular primary care outside of a medical home model	1,076,508 (10.6%)	0 (0.0%)	1,076,508 (34.9%)	902,679 (8.6%)	0 (0.0%)	902,679 (39.4%)	1,003,371 (8.7%)	0 (0.0%)	1,003,371 (38.4%)
Medical home: No regular source of primary care	2,010,451 (19.9%)	0 (0.0%)	2,010,451 (65.1%)	1,388,231 (13.2%)	0 (0.0%)	1,388,231 (60.6%)	1,612,081 (13.9%)	0 (0.0%)	1,612,081 (61.6%)
Asthma: No	8,876,591 (87.7%)	6,108,117 (86.8%)	2,768,474 (89.7%)	9,056,863 (86.0%)	7,046,703 (85.5%)	2,010,160 (87.7%)	9,791,527 (84.7%)	7,528,106 (84.2%)	2,263,421 (86.5%)
Asthma: Yes	1,248,471 (12.3%)	929,986 (13.2%)	318,485 (10.3%)	1,477,762 (14.0%)	1,197,012 (14.5%)	280,750 (12.3%)	1,768,546 (15.3%)	1,416,515 (15.8%)	352,031 (13.5%)
COPD: No	9,457,149 (93.4%)	6,508,416 (92.5%)	2,948,733 (95.5%)	9,752,325 (92.6%)	7,579,785 (91.9%)	2,172,540 (94.8%)	10,720,914 (92.7%)	8,231,267 (92.0%)	2,489,647 (95.2%)
COPD: Yes	667,913 (6.6%)	529,687 (7.5%)	138,226 (4.5%)	782,300 (7.4%)	663,930 (8.1%)	118,370 (5.2%)	839,159 (7.3%)	713,354 (8.0%)	125,805 (4.8%)
Hypertension: No	7,613,521 (75.2%)	5,027,179 (71.4%)	2,586,342 (83.8%)	7,789,296 (73.9%)	5,894,358 (71.5%)	1,894,938 (82.7%)	8,651,673 (74.8%)	6,441,172 (72.0%)	2,210,501 (84.5%)
Hypertension: Yes	2,511,541 (24.8%)	2,010,924 (28.6%)	500,617 (16.2%)	2,745,329 (26.1%)	2,349,357 (28.5%)	395,972 (17.3%)	2,908,400 (25.2%)	2,503,449 (28.0%)	404,951 (15.5%)
CHF: No	9,930,731 (98.1%)	6,882,030 (97.8%)	3,048,701 (98.8%)	10,352,281 (98.3%)	8,087,180 (98.1%)	2,265,101 (98.9%)	11,325,507 (98.0%)	8,743,192 (97.7%)	2,582,315 (98.7%)
CHF: Yes	194,331 (1.9%)	156,073 (2.2%)	38,258 (1.2%)	182,344 (1.7%)	156,535 (1.9%)	25,809 (1.1%)	234,566 (2.0%)	201,429 (2.3%)	33,13 7 (1.3%)
Diabetes: No	9,123,152 (90.1%)	6,243,941 (88.7%)	2,879,211 (93.3%)	9,323,528 (88.5%)	7,211,849 (87.5%)	2,111,679 (92.2%)	10,145,503 (87.8%)	7,739,464 (86.5%)	2,406,039 (92.0%)
Diabetes: Yes	1,001,910 (9.9%)	794,162 (11.3%)	207,748 (6.7%)	1,211,097 (11.5%)	1,031,866 (12.5%)	179,231 (7.8%)	1,414,570 (12.2%)	1,205,157 (13.5%)	209,413 (8.0%)
Dementia: No	10,040,177 (99.2%)	6,971,692 (99.1%)	3,068,485 (99.4%)	10,426,348 (99.0%)	8,152,875 (98.9%)	2,273,473 (99.2%)	11,423,059 (98.8%)	8,829,656 (98.7%)	2,593,403 (99.2%)
Dementia: Yes	84,885 (0.8%)	66,411 (0.9%)	18,474 (0.6%)	108,277 (1.0%)	90,840 (1.1%)	17,437 (0.8%)	137,014 (1.2%)	114,965 (1.3%)	22,049 (0.8%)
Mental health diagnosis in last 2 years: No	7,778,634 (76.8%)	5,215,316 (74.1%)	2,563,318 (83.0%)	8,281,506 (78.6%)	6,390,811 (77.5%)	1,890,695 (82.5%)	9,048,337 (78.3%)	6,911,069 (77.3%)	2,137,268 (81.7%)
Mental health diagnosis in last 2 years: Yes	2,346,428 (23.2%)	1,822,787 (25.9%)	523,641 (17.0%)	2,253,119 (21.4%)	1,852,904 (22.5%)	400,215 (17.5%)	2,511,736 (21.7%)	2,033,552 (22.7%)	478,184 (18.3%)

**Table 3:** Healthcare utilization by enrolment status for Ontario adults over time Percentages are row percentages. Abbreviations: ED, emergency department; ACSC, ambulatory care sensitive conditions

	Ontario Adult Population								
	2009			2014			2021		
Variable	Total	Enrolled in a medical home	Not enrolled in a medical home	Total	Enrolled in a medical home	Not enrolled in a medical home	Total	Enrolled in a medical home	Not enrolled in a medical home
Overall	10,125,062 (100%)	7,038,103 (69.5%)	3,086,959 (30.5%)	10,534,625 (100%)	8,243,715 (78.3%)	2,290,910 (21.8%)	11,560,073 (100%)	8,944,621 (77.4%)	2,615,452 (22.6%)
Any ED visits in last 2 years: No	6,716,983	4,516,370 (67.2%)	2,200,613 (32.8%)	6,861,173	5,279,120 (76.9%)	1,582,053 (23.1%)	7,807,148	5,948,631 (76.2%)	1,858,517 (23.8%)
Any ED visits in last 2 years: Yes	3,408,079	2,521,733 (74.0%)	886,346 (26.0%)	3,673,452	2,964,595 (80.7%)	708,857 (19.3%)	3,752,925	2,995,990 (79.8%)	756,935 (20.2%)
Mean number of ED visits in last 2 years among ED users (SD)	2.25 (3.00)	2.23 (2.93)	2.30 (3.21)	2.24 (2.83)	2.22 (2.68)	2.35 (3.41)	2.14 (2.92)	2.09 (2.57)	2.37 (4.01)
Median number of ED visits in last 2 years among ED users (Q1-Q3)	1 (1-2)	1 (1-2)	1 (1-2)	1 (1-3)	1 (1-3)	1 (1-3)	1 (1-2)	1 (1-2)	1 (1-3)
ACSC-related hospitalizations: No	10,096,418	7,015,404 (69.5%)	3,081,014 (30.5%)	10,504,872	8,218,900 (78.2%)	2,285,972 (21.8%)	11,534,527	8,923,782 (77.4%)	2,610,745 (22.6%)
ACSC-related hospitalizations: Yes	28,644	22,699 (79.2%)	5,945 (20.8%)	29,753	24,815 (83.4%)	4,938 (16.6%)	25,546	20,839 (81.6%)	4,707 (18.4%)
Any hospital re-admission within 30 days of discharge: No	1,976,898	1,586,935 (80.3%)	389,963 (19.7%)	1,975,702	1,689,317 (85.5%)	286,385 (14.5%)	1,887,860	1,604,344 (85.0%)	283,516 (15.0%)
Any hospital re-admission within 30 days of discharge: No	253,377	204,345 (80.6%)	49,032 (19.4%)	250,312	213,519 (85.3%)	36,793 (14.7%)	244,971	207,464 (84.7%)	37,507 (15.3%)
Any follow-up within 7 days of discharge: No	1,169,412	930,307 (79.6%)	239,105 (20.4%)	1,198,762	1,022,819 (85.3%)	175,943 (14.7%)	1,121,556	953,682 (85.0%)	167,874 (15.0%)
Any follow-up within 7 days of discharge: Yes	1,060,863	860,973 (81.2%)	199,890 (18.8%)	1,027,252	880,017 (85.7%)	147,235 (14.3%)	1,011,275	858,126 (84.9%)	153,149 (15.1%)

## Discussion

Ontario experienced improved primary care attachment rates with the introduction of medical homes, outperforming most other provinces [24,25]. However, during our study period, net gains in enrolments declined, with population-level coverage with the medical home model declining after 2015. As a result, Ontario continues to trail behind other high-income countries employing medical home models, many of which achieve near-universal primary care attachment [1,26]. The reasons likely relate to differences in the following factors.

Medical home implementation

Unlike in many other jurisdictions (e.g., Norway, Finland, the Netherlands, Denmark, the United Kingdom), Ontario’s medical home participation is neither automatic nor mandatory for either patients or physicians, and there are no fail-safes or accountability mechanisms to ensure enrolment [1]. Our results indicate this disproportionately affects vulnerable populations. Our finding that, compared to enrolled adults, unenrolled adults had a lower overall proportion of ED use but a higher proportion of ED visits among ED users may be a reflection of the diversity of the unenrolled population, such that it may include both healthier individuals with limited healthcare needs and individuals experiencing barriers to accessing continuous primary care. While healthier unenrolled adults may require little emergency care, those who do access the ED may experience more fragmented or delayed care, contributing to more frequent ED use. Policies can be adapted from peer countries to assign responsibility for universal medical home enrolment and accountabilities at all levels to ensure capacity and mitigate inequities.

Workforce supply

While our findings demonstrate increased medical home physicians per capita in Ontario, both Ontario and Canada overall have a lower number of all types of physicians per capita than international peer jurisdictions with higher primary care attachment rates [1]. As a result, family physicians cover workforce gaps in hospitals and other settings, reducing primary care capacity [1]. Longer wait times to access specialized services and tests compared with all other Commonwealth Fund Study countries further strain capacity, as patients need management in primary care while they wait [26].

Remuneration and infrastructure

Countries with high medical home attachment use capitation or salary models with interprofessional teams [1]. Ontario, however, began restricting these structures within 10 years of launching the medical home model [20], with the strongest restrictions beginning in 2015 [19] - a point after which our data show a decline in the proportion of adults attached to a medical home. Demand for family physicians in other, more specialized settings, combined with better remuneration and work conditions in such settings compared with fee-for-service primary care, may explain why more medical home physicians per capita, a trend that we show continued beyond 2015, has not translated to increased enrolment rates [1,27]. In other words, with policies restricting entry into the higher remunerated capitation-based medical homes and interdisciplinary teams, family physicians able to enter only enhanced fee-for-service, non-interdisciplinary medical homes may be concurrently drawn to practice opportunities outside their medical home, reducing enrolment capacity. Expanding preferred models of care will likely be needed in many Canadian and international contexts to attract and retain primary care physicians at a time of a critical primary care workforce shortage.

In effect, Ontario’s medical home model was incompletely implemented, limiting its impact at the population and health system levels. In February 2024, the province began implementing new investments into the medical home model, culminating in the introduction of its Primary Care Action Plan in January 2025, which expands interdisciplinary teams, and, as of April 1, 2026, the implementation of a new physician services agreement that allows more physicians to enter capitation-based medical homes. These developments align with the insights from our findings. Likewise, other Canadian and international jurisdictions may draw from Ontario’s medical home experience as they pursue reforms to improve primary care attachment.

Limitations

Aside from patients who died or moved out of the province during the study period (and were therefore excluded), it is difficult to determine why patients lost their enrolment in a medical home or whether de-enrolment was initiated by the patient or physician. Termination codes can be submitted by physicians to the Ministry of Health for administrative purposes, but our analysis found these codes unreliable. Improved accuracy in recording de-enrolment reasons may inform targeted interventions. We likewise cannot confirm the reasons underlying the major spikes (2015) and dips (2020) in net enrolments and medical home physicians per capita, though we hypothesize these relate to impending restrictions on medical home entry for physicians (2015), prompting a surge in uptake of medical home model, and the onset of the COVID-19 pandemic (2020), prompting a decline in the workforce and its capacity to enroll more patients [28]. Patients not in a medical home are not captured in our analyses, yet these patients may still have a regular primary care provider [29]. That said, previous research indicates that informal primary care attachment may be insufficient to support important population-level performance measurement and quality improvement [16,30].

Patients who visited a community health center or community health group could not be included; however, these models serve less than 2% of the overall Ontario population [29]. Unattachment duration is truncated by the study period, potentially underestimating true durations. Although our results appear to coincide with policy changes affecting physician access to certain types of primary care medical homes, this study is observational and therefore cannot be used to infer causality. Subgroup differences in enrolment patterns should be interpreted descriptively, as analyses were unadjusted. As with other studies using health administrative data, our findings are subject to limitations related to potential misclassification and the limited breadth of variables captured within these datasets. Finally, the analyses do not include data after 2021. This reflects the lag in the availability of health administrative data, as well as the study team’s intention to establish cohorts of patients gaining or losing medical home enrolment for future longitudinal analyses requiring a minimum two-year follow-up period. As a result, the findings do not capture changes in primary care attachment beyond 2021, although between 2021 and 2024, no significant new policies related to medical homes were introduced in Ontario.

## Conclusions

Policies limiting the entry of family physicians into capitation-based and interdisciplinary team-based medical homes coincided with an erosion in prior gains made to medical home enrolments among Ontario adults, despite an increase in per capita family physician supply. The analyses presented here provide a foundation for future work analyzing the impacts of gaining or losing primary care medical home enrolment on health system costs and the utilization of other sources of care, such as hospitals and EDs.

## Figures and Tables

**Figure 5:**
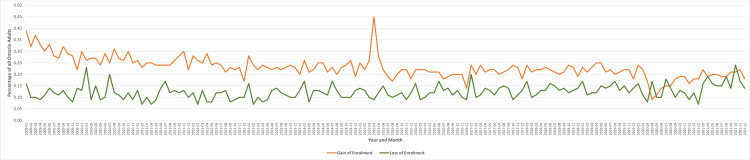
Monthly gains and losses in enrolment over time

**Figure 6:**
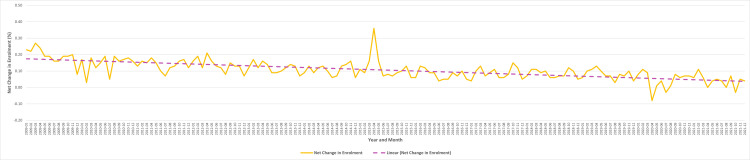
Net changes in enrolment over time

**Figure 7:**
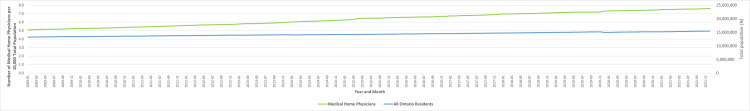
Medical home physicians per capita over time compared with population growth

**Figure 8:**
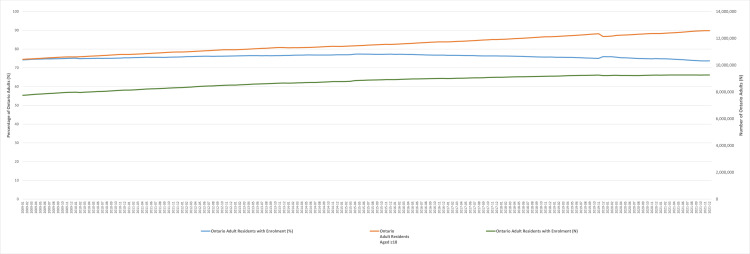
Ontario adults enrolled in a medical home over time (% and N) compared with population growth (N)

## Appendices

Appendix A: Data sources and covariate definitions

Data were sourced from the Registered Persons Database (RPDB) (patient-level age, sex, postal code, and newcomer status), Ontario Marginalization Index dataset (patient-level economic, social, and ethno-racial marginalization), Primary Care Population (PCPOP) dataset (patient-level core primary care visits, hospitalizations, ED visits, chronic diseases, and medical home model), and Client Agency Program Enrolment (CAPE) database (physician-level medical home model). Patient comorbidities and health utilization were measured with the Aggregated Diagnostic Groups (ADGs) and Resource Utilization Bands (RUBs) based on the Johns Hopkins Adjusted Clinical Grouping (ACG) system v10.0.1 (build 879), which is a patient-level diagnosis-based method using inpatient and outpatient administrative data from the Canadian Institute for Health Information Discharge Abstract Database (DAD) (inpatient hospitalizations), Same Day Surgery (SDS) (outpatient surgeries), National Ambulatory Care Reporting System (ED visits) and Ontario Health Insurance Plan (OHIP)(health services claims) datasets.

Appendix B: Expanded figures
